# The Plasma Membrane at the Cornerstone Between Flexibility and Adaptability: Implications for *Saccharomyces cerevisiae* as a Cell Factory

**DOI:** 10.3389/fmicb.2021.715891

**Published:** 2021-08-09

**Authors:** Luís Ferraz, Michael Sauer, Maria João Sousa, Paola Branduardi

**Affiliations:** ^1^Center of Molecular and Environmental Biology, University of Minho, Braga, Portugal; ^2^Department of Biotechnology and Biosciences, University of Milano Bicocca, Milan, Italy; ^3^Department of Biotechnology, Institute of Microbiology and Microbial Biotechnology, BOKU University of Natural Resources and Life Sciences, Vienna, Austria

**Keywords:** plasma membrane, yeast, membrane engineering, microbial cell factories, robustness, lipids

## Abstract

In the last decade, microbial-based biotechnological processes are paving the way toward sustainability as they implemented the use of renewable feedstocks. Nonetheless, the viability and competitiveness of these processes are often limited due to harsh conditions such as: the presence of feedstock-derived inhibitors including weak acids, non-uniform nature of the substrates, osmotic pressure, high temperature, extreme pH. These factors are detrimental for microbial cell factories as a whole, but more specifically the impact on the cell’s membrane is often overlooked. The plasma membrane is a complex system involved in major biological processes, including establishing and maintaining transmembrane gradients, controlling uptake and secretion, intercellular and intracellular communication, cell to cell recognition and cell’s physical protection. Therefore, when designing strategies for the development of versatile, robust and efficient cell factories ready to tackle the harshness of industrial processes while delivering high values of yield, titer and productivity, the plasma membrane has to be considered. Plasma membrane composition comprises diverse macromolecules and it is not constant, as cells adapt it according to the surrounding environment. Remarkably, membrane-specific traits are emerging properties of the system and therefore it is not trivial to predict which membrane composition is advantageous under certain conditions. This review includes an overview of membrane engineering strategies applied to *Saccharomyces cerevisiae* to enhance its fitness under industrially relevant conditions as well as strategies to increase microbial production of the metabolites of interest.

## Introduction

The urge for the production of goods and services for a growing population, adopting the principles of linear economy, has led to massive consumption of natural resources, resulting in an unbalance between the request and the supply of these resources. Biorefineries are intended to address this urgency by utilizing and converting residual and renewable biomasses into a spectrum of marketable products such as biofuels, materials, chemicals, feed, and food (Branduardi, 2021). Among the diverse methods of valorization of biomass, microbial fermentations offer many possibilities for obtaining the desired products. The diverse nature of the feedstocks, together with the biodiversity of microorganisms, has the potential to lead to the production of many classes of products (Hong and Nielsen, 2012). Yeasts are among the most prominent microorganisms used in industrial biotechnology because they unify the advantage of unicellular organisms with eukaryotic nature. *Saccharomyces cerevisiae*, in particular, plays a major role, mainly thanks to its ancient history of domestication by humans (Branduardi and Porro, 2012). Moreover, microbial biotechnology applications have increased in the last decades, with the constant evolution of genomics, metabolic engineering, systems, and synthetic biology. This has enabled the production of numerous valuable products of primary and secondary metabolism, enzymes and biopharmaceutical proteins, which are of high demand in various industrial sectors. Once more, *S. cerevisiae* is still scoring positive returns as a cell factory (Li and Borodina, 2015).

Nonetheless, harsh industrial conditions put microbial cell factories in very stressful environments. The development of tolerant strains, able to handle the requirements of industrial processes is therefore highly desirable. During industrial processes, microorganisms can be subjected to many and different kinds of stresses: high metabolite concentration, substrate variety, high osmotic pressure and ion toxicity, high temperature, extreme pH, high concentrations of weak acids, among others that compromise cell metabolism (Deparis et al., 2017). In this regard, the plasma membrane plays a key role since it is a physical barrier that separates the extracellular environment and intracellular components, is responsible for maintaining the correct ion homeostasis and is the sensor of the overall cellular environment, rearranging its composition in response to different stimuli (Stewart, 2017). Moreover, increased production of metabolites of interest by the microbial cell factory also puts augmented pressure on the plasma membrane. It is therefore evident that the plasma membrane is crucial for the successful development of many bioprocesses (Jezierska and Van Bogaert, 2017).

Thus, the plasma membrane has to be considered when designing strategies for the development of versatile, robust and efficient cell factories ready to tackle the harshness of industrial processes while delivering high yield, titer and productivity. In this sense, the concept of membrane engineering has emerged. The plasma membrane is a complex and dynamic system, whose behavior is challenging to predict due to the connections, competitions, dependencies, or other types of interactions between its components. Furthermore, the behavior of a system is also influenced by its surrounding environment. This is precisely what happens at the membrane level, where lipids and proteins interact and influence each other. For this reason, it is not trivial to predict which element(s) should be changed to trigger a specific rewiring of the overall system.

When employing membrane engineering strategies, researchers have mainly been focusing on alterations in single elements of the plasma membrane rather than on the membrane as a whole system. Changes in single elements might trigger an overall response of the membrane resulting in a global reshaping of the system. On the other hand, being a highly interconnected network, the plasma membrane might not be affected by single modifications as it can counteract minor alterations by different regulation mechanisms (Sandoval and Papoutsakis, 2016; Jezierska and Van Bogaert, 2017).

This review aims to describe the most recent efforts to engineer the plasma membrane of microbial cell factories, with particular emphasis on *S. cerevisiae*, to increase its fitness and performance in biotechnological processes.

## Yeast Plasma Membrane

All cells are surrounded by a plasma membrane that defines the boundary between the cell itself and the environment. In *S. cerevisiae*, the cell envelope alone (plasma membrane and cell wall) occupies about 15% of the total cell volume (Stillwell, 2016).

In bacteria and eukaryotic cells, the plasma membrane is structured as a bilayer mainly composed of a mixture of (phospho)lipids and proteins which, by their interactions, govern the structure of the membrane and determine its physicochemical properties.

Lipids represent around 50% of the membrane composition. The major lipid classes present in the *S. cerevisiae* membrane are glycerophospholipids (about 70%), sphingolipids (about 15%) and sterols (about 15%) (Table 1; Klose et al., 2012). Glycerophospholipids have a glycerol backbone connected to two fatty acyl chains by an ester-linkage and can be further divided according to their head group, attached to glycerol through phosphate, into: phosphatidylcholine (PC), phosphatidylethanolamine (PE), phosphatidylinositol (PI), phosphatidylserine (PS), phosphatidylglycerol (PG), and phosphatidic acid (PA) (Klug and Daum, 2014). The fatty acyl chains of glycerophospholipids are usually C_16_–C_18_. Sphingolipids contain a sphingoid backbone connected by an amide link to a fatty acyl chain and are classified by their head group into inositol phosphoryl ceramide (IPC), mannosyl-inositol phosphoryl ceramide (MIPC) and mannosyl-di-inositol phosphoryl ceramide (MIP_2_C). Sphingolipids have long-chain bases (LCB) originating from C_16_–C_18_ fatty acids combined with very-long-chain fatty acid (VLCFA), usually C_24_–C_26_. Sphingolipids acyl chains are completely saturated, differently, the acyl chains of glycerophospholipids can be unsaturated (Megyeri et al., 2016). Saturated fatty acyl chains increase lipid packing and membrane thickness due to their straight conformation, in comparison to unsaturated fatty acyl chains, which have a bent conformation. Furthermore, long fatty acyl chains increase membrane rigidity by increasing the membrane thickness and lipid packaging (Slotte, 2016). Ergosterol is the main sterol in yeast: its general structure comprises a hydroxyl head group, an alkenyl side chain and a four-ring nucleus (Klug and Daum, 2014). Sterols increase membrane rigidity by ordering fatty acyl chains allowing tighter lipid packing (Caron et al., 2014). Nonetheless, portraying the impact of lipid species on the membrane physicochemical properties is an oversimplification and the reality is not as straightforward. The lipid composition of membranes is not constant. In *S. cerevisiae*, the ratios of lipids may differ not only among different strains but also depending on the carbon source and cultivation conditions (Patton and Lester, 1991). Changes in the lipid composition can have profound effects on cellular functions, including signal transduction, membrane elasticity, and membrane trafficking (Santos and Preta, 2018). Moreover, the biosynthesis pathways leading to different lipid species are tightly connected with complex cross-talks (Henry et al., 2012).

**TABLE 1:** Main classes and subclasses of lipids in *S. cerevisiae*.

Lipid classes	Lipid subclasses
Glycerophospholipids	Phosphatidylcholine (PC)
	Phosphatidylethanolamine (PE)
	Phosphatidylinositol (PI)
	Phosphatidylserine (PS)
	Phosphatidylglycerol (PG)
	Phosphatidic acid (PA)
Sphingolipids	Ceramide (CER)
	Inositol phosphoryl ceramide (IPC)
	Mannosyl-inositol phosphoryl ceramide (MIPC)
	Mannosyl-di-inositol phosphoryl ceramide (MIP_2_C)
Sterols	Ergosterol

Another major component of the plasma membrane is proteins, representing around 40% of the membrane composition. A total of 1,000 different proteins are estimated to be located in the yeast plasma membrane (Stewart, 2017). Not all of these proteins are present at the same time and the amount and type of proteins changes according to different cellular stimuli, which means that the actual number of functional membrane proteins is much smaller. The majority are transport proteins while other membrane proteins are involved in cell wall synthesis, signal transduction or take part in the definition of the cytoskeleton. Remarkably, the activity and stability of membrane proteins is dependent on the lipids that surround them (Coskun and Simons, 2011). Membrane proteins require specific lipids, as cofactors for their functions or as “co-structures” for their correct folding and stability. Therefore, the composition of the lipid bilayer must be optimal for obtaining the correct activity or the desired biological function of the membrane proteins (Lee, 2004). This awareness is very important (and far from being trivial) when introducing heterologous proteins in different microorganisms (Opekarová and Tanner, 2003). Also, the physicochemical properties of the plasma membrane, such as thickness, viscosity, tension and permeability are affected by the interactions between lipids and proteins (Lee, 2004). Lipid rafts are a good example of the interaction between lipids and proteins (Klose et al., 2010). Lipid rafts are dynamic nanoscale ergosterol and sphingolipid-enriched clusters with higher order than the surrounding membrane areas. It has been suggested that the bulky sterol rings pack better next to saturated acyl chains of sphingolipids and are shielded from the aqueous environment by the large sphingolipids head groups. Membrane rafts house several membrane proteins as some concentrate in these specific areas, among which, the proton pump H + -ATPase Pma1 (Ferreira et al., 2001). This is the major proton pump present in the yeast plasma membrane and represents 15% of all plasma membrane proteins. The importance of this proton pump for yeast cells under different stress conditions has been highlighted [see for a recent example Lee et al. (2017)]. The correct positioning and activity of Pma1 into plasma membrane lipid rafts has been correlated with the presence of very long chain fatty acids and ergosterol (Eisenkolb et al., 2002; Gaigg et al., 2006). These studies highlighted the importance of membrane lipid composition for the correct integration and functioning of proteins in the plasma membrane.

## Exploring Microbial Membranes

It becomes evident that either for understanding biological functions or for exploiting cellular systems in microbial-based processes, biochemical and physical understanding of plasma membranes is essential. Nevertheless, it is difficult to predict which membrane composition will lead to a specific emerging property. However, it is possible to use and integrate the data deriving from different research technologies (Table 2) to describe the plasma membrane not as a sum of elements, but as a whole system.

**TABLE 2:** Overview of research techniques used to characterize different parameters of the plasma membrane.

Technique	Strengths	Limitations	Relevant works cited within this review
Lipidomics	Identification and quantification of lipids	Accuracy	Klose and Tarasov (2016)
Molecular dynamic simulations	Physiochemical characterization of the plasma membrane	Simplicity of the models available	Pluhackova and Böckmann (2015)
Atomic force microcopy	Working on living cells under physiological relevant conditions, Investigation of cell surface nanomechanical properties	Sample preparation, Scanning speed, Low scan image size	Dufrêne (2002), Francois et al. (2013), Shi et al. (2018)
*In vitro* membrane models	Enables the prediction of physiochemical properties of lipid bilayers	Limited to the classes of lipids commercially available	Lopes et al. (2017)
Genomic scale metabolic models (GEMs)	Helpful to predict phenotypes based on genotype manipulation.	Reproducibility of simulations “*in vivo*” is dependent on the quality of the GEM	Garcia-Albornoz and Nielsen (2013)

Lipids are highly complex and dynamic molecules with thousands of species dynamically changing to support variations in physiological and environmental conditions. For this reason, the identification and quantification of lipids over time is very difficult (Yang and Han, 2016). Lipidomics is the technology that aims to analyze and quantify lipid species in a cell, organism or context (Klose and Tarasov, 2016). Lipidomics methods reveal the lipid status of a cellular phenotype at a particular time point and therefore allow researchers to correlate a specific membrane lipid composition with specific conditions. However, it does not allow researchers to understand the cellular mechanism which led to a specific membrane composition.

To obtain further information related to the structure and the dynamics of the plasma membrane molecular dynamics (MD) simulations are used (Pluhackova and Böckmann, 2015). MD simulation studies can be used to investigate the physicochemical properties of the membrane, the interdependent influence of proteins and lipids and also the formation of membrane nanodomains (Lindahl et al., 2016, 2018). MD simulations may also be used as predictive, to provide strategies for membrane engineering. However, most MD simulations are performed using very simple membrane models which limits the biological relevance of these studies.

Atomic Force Microscopy (AFM) is another valuable tool used in membrane research. AFM has emerged as a powerful tool to investigate microbial cells at the nanoscale level and to measure the nanomechanical properties of cell surface topology such as stiffness, elasticity or roughness (Dague et al., 2010; Shi et al., 2018). AFM provides three-dimensional views of biological structures in real-time, on living cells and also under biologically relevant conditions (Alsteens et al., 2017). AFM can provide complementary information on how cells can cope with a certain type of stress, which can be relevant in the design of novel strategies for the development of improved microbial cell factories. Using AFM, Niu et al. (2016) studied the effect of ethanol stress on *S. cerevisiae* plasma membrane elasticity and fluidity. Under ethanol stress, the integrity of the plasma membrane was reduced which led an increased membrane permeability and fluidity. Furthermore, an increase on the plasma membrane elasticity was also observed. For a more detailed information on the application of AFM to explore yeast cells under stress conditions the reader should look at the following manuscripts (Dufrêne, 2002; Francois et al., 2013; Schiavone et al., 2016).

*In vitro* membrane models represent another way to study the physicochemical properties of lipid bilayers (Lopes et al., 2017). However, these models rely on commercially available lipids. There is still a lack of internal standards for some lipids classes and therefore, these types of studies are usually done using very simple membrane models consisting of only some classes of lipids. For these reasons, the physiological relevance of these studies is often limited.

Genome-scale metabolic models (GEMs) can also be a valuable tool for the design and optimization of microbial cell factories. The reproducibility of the simulations in “*in vivo*” data is, however, dependent on the quality of the GEM (Garcia-Albornoz and Nielsen, 2013). Recently, Tsouka and Hatzimanikatis (2020) developed a metabolic model called “Reduced lipids-centric model” (redLips), which focused on the lipid metabolism of *S. cerevisiae*. “RedLips” was constructed through the integration of detailed lipid metabolic pathways into already existing genome-scale metabolic models. Overall, this model can be used as a scaffold for integrating lipidomics data to improve predictions in studies of lipid-related biological functions (Tsouka and Hatzimanikatis, 2020).

## Membrane Engineering in Microbial Workhorses

**FIGURE 1:**
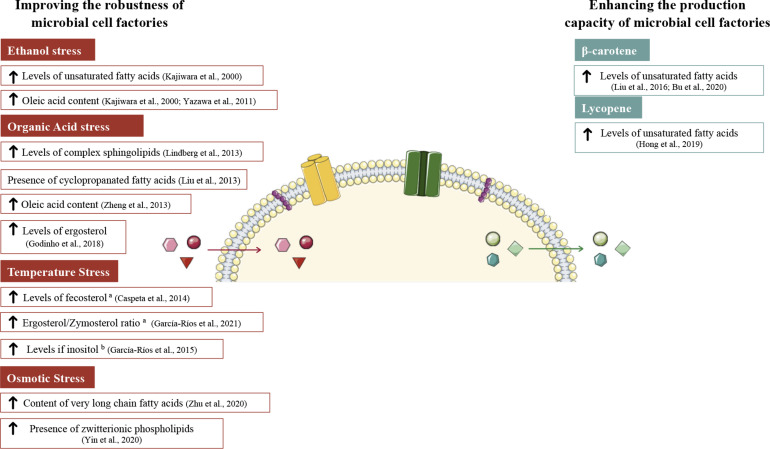
Plasma membrane modifications in *S. cerevisiae* aiming to improve yeast fitness on industrial bio-processes. On the left side are represented modifications to increase yeast robustness toward stress conditions (ethanol, organic acids, temperature, and osmotic stress). Plasma membrane modifications on the right side were done to enhance the production capacity of microbial cell factories. Red arrows indicate the entrance of the stress agents. Green arrows indicate the exit of metabolites of interest. ^a^ and ^b^ stand for works performed at high and low temperatures, respectively. The figure was produced using the vector image bank of Servier Medical Art (http://smart.servier.com/).

Overall, the different technologies that allow researchers to study the plasma membrane are still incomplete in describing what is occurring at the membrane level at a certain time point, and even more in the fluctuating conditions of industrial processes. Notably, several approaches involving the plasma membrane have been used to ameliorate yeast cell factories. In this section, we provide an overview of membrane engineering strategies applied to *S. cerevisiae* to improve its robustness toward different stress conditions present at industrial levels, strategies focusing on improving *S. cerevisiae* productivity, yield and production will also be described (Figure 1).

### Improving the Robustness of Microbial Cell Factories

Robustness is an important beneficial trait for any microorganism to acquire improved fitness, which in some cases correlates with an efficient biosynthesis of desirable molecules (Nicolaou et al., 2010). During biotechnological processes, yeasts encounter different kinds of stresses that are generally associated with the growth conditions (temperature, pH, oxygenation, …), the starting substrates and the final products or byproducts (Gibson et al., 2007). Because the economic viability of bioprocesses is often limited by damage to the microbial plasma membrane, assessing changes due to stressors becomes essential to design counteractions.

#### Ethanol

The effects of ethanol on *S. cerevisiae* membrane permeability were firstly described by Leão and Van Uden (1984), who reported an increased permeability of the plasma membrane to protons in the presence of ethanol. Later it was shown that ethanol also increases the diffusion of acetic acid in *S. cerevisiae* (Casal et al., 1998). More recently, a similar effect was also reported by Niu et al. (2016), who showed that the exposure of yeast cells to ethanol resulted in increased membrane permeability (characterized by relative electric conductivity) together with an increase in cell swelling rate, indicating that the plasma membrane integrity was reduced in the presence of ethanol. Furthermore, using AFM, Schiavone et al. (2016) demonstrated that ethanol stress caused a reduction of the plasma membrane thickness.

The relationship between ethanol tolerance and lipid composition of the plasma membrane is strongly dependent on the experimental conditions that are used. For this reason, a clear connection between plasma membrane composition and ethanol tolerance remains to be elucidated (Henderson and Block, 2014).

A common strategy to improved *S. cerevisiae* robustness to ethanol has been the development of strains with an increased fraction of glycerophospholipids containing oleic acid (C_18__:__1_). Oleic acid has unique properties: on one side its 18 carbon chain length contributes to increasing membrane thickness, but on the other side its degree of unsaturation contributes to an increase of membrane fluidity and decreases membrane thickness. The effect of increased amounts of oleic acid in the plasma membrane is difficult to predict and must be verified experimentally case by case, considering the overall changes in fatty acyl chain length and saturation, and the experimental conditions. Kajiwara et al. (2000) reported a 33% increase in oleic acid content at the expense of palmitic acid (C_16__:__0_) when *OLE1*, encoding for a stearoyl-CoA 9-desaturase which catalyzes the transformation of saturated fatty acids (C_16__:__0_ and C_18__:__0_) to unsaturated fatty acids (C_16__:__1_ and C_18__:__1_), was overexpressed in *S. cerevisiae*, overall measuring a 7% increase in unsaturated fatty acids. The engineered strain showed improved growth and ethanol production at low temperatures (10°C). Since lowering of the temperature leads to a more ordered membrane structure and hence a reduction in fluidity (Shinitzky and Henkart, 1979) which is largely determined by the packing of oleic acid (C_18__:__1_) and palmitoleic acid (C_16__:__1_), the impact of the observed increase in oleic acid in membrane fluidity could potentially underlay the improved performance of the strain.

Differently, in other study, conducted at 30°C, the overexpression of *OLE1* did not result in changes in the content of oleic acid (Zheng et al., 2013). This discrepancy might be because cells increase their membrane unsaturation at lower temperatures (Aguilera et al., 2007).

*Saccharomyces cerevisiae* membrane engineering can also be based on the introduction of heterologous genes or lipid species. Yazawa et al. (2011) expressed the rat elongase 1 gene (*rELO1*) in *S. cerevisiae*, obtaining vaccenic acid instead of oleic acid, but with no effect on ethanol tolerance. The desired effect was reached by the introduction of rat elongase 2 (*rELO2*), which increased oleic acid content by 18%. Importantly, under these conditions the unsaturation index was very similar to that of the control, meaning that the increase of oleic acid was determinant for ethanol tolerance. Furthermore, *rELO2* overexpression also conferred tolerance to n-butanol, n-propanol, and 2-propanol.

Despite the positive results obtained, it is not clear if the advantage derives from the oleic acid content or other alterations affecting the membrane properties, as they were not measured. Indeed, other attempts seem to suggest that we are still exploring trial and error strategies.

#### Organic Acids

The effects of weak organic acids on *S. cerevisiae* have been generally ascribed to acidification of the cytosol by protons released and/or accumulation of the anionic form of the acid. These, in turn, can cause several cell alterations such as the disruption of the proton gradient across the plasma membrane, increased turgor pressure, oxidative stress, protein aggregation, lipid peroxidation, inhibition of membrane trafficking, and perturbation of plasma and vacuolar membranes spatial organization (Piper, 2011).

Guo et al. (2018) performed a comparative analysis of lipids in *S. cerevisiae* grown in the presence of four different weak acids, which are known to be detrimental for yeast growth: acetic, cinnamic, formic, and levulinic acids.

Yeast cells counteract the stress caused by organic acids by modulating the plasma membrane lipid composition and the modifications differ depending on the molecular conformations of the acids. Formic, levulinic and acetic acids did not affect the levels of PA in comparison to the control, while in cells exposed to cinnamic acid there was a decrease of 30% in PA content. Furthermore, the contents of PE, PC, and PS decreased in yeast cells under any acid condition compared to the control. Differently, the content of PI increased in yeast cells subjected to acid stress during growth on glucose, but decreased during the adaptation phase on ethanol, compared to the control. Concerning the fatty acids (profile FAs), in yeast cells under acid stress, the amount of myristic acid (C_14__:__0_) was around 5% of the total FAs, similar to that of the control. However, a decrease in palmitic acid (C_16__:__0_) and palmitoleic acid (C_16__:__1_) and an increase in elaidic acid (C_18__:__1_) and stearic acid (C_18__:__0_) were observed for all the acid-stressed cells. While the unsaturation index in cells exposed to cinnamic acid was unaffected, exposure to acetic, formic and levulinic acid resulted in an increase in the saturation index of FAs in comparison to the control. Exposure of the cells to acids led to a continuous increase of the sterol content during the different phases of growth, while the control showed a gradual decrease in ergosterol from the exponential phase to the stationary phase. Ergosterol, the major yeast sterol, is essential for the structure of the plasma membrane, modulating its thickness, fluidity and permeability and regulating the activity of membrane-associated transporters (Abe and Hiraki, 2009; Kodedová and Sychrová, 2015). Therefore, ergosterol plays a key role in the resistance toward inhibitory compounds.

The work developed by Guo et al. (2018) has the important merit to describe how the plasma membrane lipid composition adapt to different stress factors and could be used as a guide for membrane engineering strategies, despite it did not take into account the membrane physicochemical properties as well as the rearrangement of membrane proteins.

Lindberg et al. (2013) explored a comparative approach: in an attempt to increase *S. cerevisiae* tolerance to acetic acid, the authors studied the lipid profile of *Zygosaccharomyces bailii*, a food spoilage yeast well known for its resistance to weak organic acids (Fleet, 1992). After exposure to acetic acid, *Z. bailii* revealed large lipidomic changes while smaller changes were observed in *S. cerevisiae*. A higher degree of saturation of the glycerophospholipids and increased amounts of complex sphingolipids at the expense of glycerophospholipids were the most noticeable changes in the adaption of *Z. bailii* to acetic acid. These results are consistent with the previously described role of sphingolipids in cell death induced by acetic acid in *S. cerevisiae*, where the deletion of *ISC1*, coding for the inositol phosphosphingolipid phospholipase C, responsible for the hydrolysis of complex sphingolipids, lead to increased resistance to acetic acid (Rego et al., 2012).

By combining lipidomic analysis with molecular dynamic simulations, in a multidisciplinary work, Lindahl et al. (2016) reported that membranes with high content of sphingolipids are thicker and denser than control and membrane permeability decreases. Taking this into account, Lindahl et al. (2017) tried to increase the fraction of complex sphingolipids in the plasma membrane of *S. cerevisiae* by altering the expression of genes associated with the production of long-chain base (LCB) and very-long-chain fatty acids (VLCFA) (C_24__–__26_), and with the conversion of ceramides into complex sphingolipids. The authors overexpressed *ELO3*, involved in fatty acid elongation and *AUR1*, encoding an enzyme that catalyzes the formation of complex sphingolipids, and deleted *ORM1* and *ORM2*, encoding negative regulators of sphingolipids biosynthesis. However, neither the overexpression of *ELO3* or *AUR1* influenced the lipid metabolism. The deletion of *ORM1* and *ORM2* lead to a decrease in both complex sphingolipids and phosphatidylinositol, which diminished cell viability. When combined, the reduction in growth caused by the *orm1/2* deletion was alleviated by the overexpression of *ELO3* and *AUR1*, which also determined an increase in the fatty acyl chain length. Overall, the authors were not successful in the attempt to increase levels of complex sphingolipids in *S. cerevisiae*.

Increasing the length of fatty acids has been a common strategy employed to increase microorganisms’ fitness toward organic acids. Zheng et al. (2013) overexpressed *ELO1*, encoding a fatty acid elongase, in *S. cerevisiae* to improve cellular tolerance to acetic acid. These authors observed an 18% increase in the cellular content of oleic acid, which resulted in a 44% increase in survival after acetic acid exposure, but the molecular system was not completely described.

Godinho et al. (2018) unveiled the relation between the yeast ABC transporter Pdr18 and ergosterol levels in yeast adaptation and tolerance to acetic acid stress. Pdr18 has been proposed to mediate the incorporation of ergosterol in the plasma membrane. The authors reported a coordinated activation of the transcription of Pdr18 and several ergosterol biosynthesis pathway genes during the period of adaptation to acetic acid. Therefore, Pdr18 has been suggested to be essential to keep maximum ergosterol content in the plasma membrane in the presence of acetic acid, thus maintaining the plasma membrane order, electrochemical potential and permeability in the presence of acetic acid. The role of Pdr18 in the maintenance of the plasma membrane physicochemical properties in the presence of acetic acid is crucial for the adequate functioning of the membrane.

The expression of heterologous genes in *S. cerevisiae* to increase its robustness to organic acids has also been attempted. Cyclopropane ring formation on fatty acyl chains occurs in both bacteria and archaea and it has been associated with an increase in the plasma membrane rigidity (Oger and Cario, 2013). Liu et al. (2013) expressed *Escherichia coli cfa* gene in *S. cerevisiae* and successfully converted 10% of fatty acids into cyclopropanated fatty acids. However, mutants failed to show octanoic acid resistance and no further stress conditions were tested.

#### Thermotolerance

Thermotolerance is a desirable trait in microbial cell factories: it can result in reduced cooling costs, and contamination risks and can boost enzyme activity during simultaneous saccharification and fermentation (Abdel-Banat et al., 2010). However, heat is also a stress factor, known to disturb protein stability, cell membrane order, and cytoskeleton structures, with consequences such as protein dysfunction, metabolic imbalances and loss of metabolic activity (Verghese et al., 2012).

Caspeta et al. (2014) set up an adaptive laboratory evolution (ALE) experiment to select yeast strains with improved growth and ethanol production at temperatures higher than 40°C. Sequencing of the evolved strain, capable to grow at high temperatures, revealed, among other findings, a point mutation in the *ERG3* gene, encoding a structural enzyme in the ergosterol biosynthesis pathway. In this strain, fecosterol, a sterol precursor, became the major sterol in the plasma membrane rather than ergosterol. The authors reported that the substitution of the “flat” ergosterol by the “bended” fecosterol in the evolved *S. cerevisiae* strain seems to be responsible for the maintenance of optimal membrane fluidity at high temperatures and therefore crucial for the thermotolerant phenotype (Caspeta et al., 2014).

More recently, García-Ríos et al. (2021) aimed to generate a robust *S. cerevisiae* strain to be used in cocoa fermentation. ALE was conducted in a defined medium at 40°C for 150 generations. The evolved strain exhibited a significantly increased growth rate in comparison to the parental strain. Lipidomic analysis revealed that, at 40°C, the evolved strain exhibited a higher ergosterol/zymosterol ratio compared to the parental strain. Authors claim that this difference could be responsible for the adaptation of the evolved strain to higher temperatures. Differently from the higher levels of fecosterol reported by Caspeta et al. (2014), in the obtained evolved strain García-Ríos et al. (2021) observed the accumulation of episterol, which is the next intermediate in the sterol biosynthesis pathway. Furthermore, no significant differences were found in terms of the fatty acid profile between the two strains.

These results highlight that the adaptation of the yeast plasma membrane to heat stress is not straightforward and membrane lipid composition changes differently according to the background of the strain used and also with the conditions in which the ALE experiments are carried out.

In another work, Liu et al. (2017) compared the thermotolerance of *erg2*Δ, *erg3*Δ, *erg4*Δ, *erg5*Δ, *erg3*Δ*erg4*Δ, *erg3*Δ*erg5*Δ, and *erg4*Δ*erg5*Δ *S. cerevisiae* strains. The mutants lacking either of the four enzymes are viable, with intermediate sterols instead of ergosterol accumulated in the membrane. All mutant strains displayed a higher growth rate than wild type at 39.5°C. The *erg3*Δ*erg5*Δ strain, in particular, exhibited and 2.24-fold increase in growth rate relative to wild type at this temperature (Liu et al., 2017). Modifications of the sterol composition directly affect the fluidity and permeability of the plasma membrane, as well as the localization and activity of membrane proteins (Kodedová and Sychrová, 2015). This work highlighted the importance of the sterol composition on yeast response to high temperatures and can be used as a guide for future membrane engineering approaches.

Different from these approaches, the study of the membrane composition of thermotolerant yeasts such as *Kluyveromyces marxianus* can be valuable to provide guidelines and ideas to engineering the plasma membrane of *S. cerevisiae*.

Temperature is also one of the most important parameters affecting wine fermentation. Low fermentation temperature improves the characteristic taste and aroma of wines. However, low temperature fermentations result in increased lag phases and lower growth rates for yeasts, causing fermentation to stop (Bisson, 1999). As mentioned above, low temperatures also affect the plasma membrane, leading to a decrease in membrane fluidity (Shinitzky and Henkart, 1979). Metabolic profiling done by López-Malo et al. (2013b) revealed that the main metabolic differences between *S. cerevisiae* growing at 12°C (common fermentation temperature for wine) and 28°C were related to lipid metabolism.

Genes involved in the phospholipid, sphingolipid and ergosterol metabolism were identified as those causing the most significant effects on yeast growth at low temperatures (López-Malo et al., 2013a). *OLE1* was one of the genes identified and its overexpression lead to an improved fermentation ability at lower temperatures (12°C) in synthetic must. Furthermore, the wine produced from this strain revealed a specific aroma profile (López-Malo et al., 2014). As previously mentioned, *OLE1* encodes a stearoyl-CoA 9-desaturase which catalyzes the transformation of saturated fatty acids (C16:0 and C18:0) to unsaturated fatty acids (C16:1 and C18:1). The presence of unsaturated fatty acids contributes to increased membrane fluidity, which is advantageous for the cells at low temperatures (Aguilera et al., 2007).

In a different approach, López-Malo et al. (2015) performed an ALE in synthetic must to obtain a wine yeast strain able to ferment at low temperatures. The evolved strain exhibited improved growth and higher fermentation performance at low-temperature in comparison to the parental strain. Genome sequencing of the evolved strain revealed the presence of a single nucleotide polymorphism (SNP) in the *GAA1* gene, which encodes a subunit of the glycosylphosphatidylinositol (GPI) transamidase complex. This complex adds GPI, required for inositol synthesis, to newly synthesized proteins, including mannoproteins. Inositol is an essential phospholipid precursor in yeast cells and could be incorporated into phosphatidylinositol (PI), sphingolipids and glycosylphosphatidylinositol anchors. Using a reverse engineering strategy, a site-directed mutation (*GAA1*Thr108) was introduced in the parental strain, which resulted in improved fermentation performances. This result reveals a higher inositol requirement for *S. cerevisiae* cells grown at low temperatures.

Overall, these works highlighted the importance of the plasma membrane lipid composition in yeast response to sub-optimal temperatures.

#### Osmotic Stress

Osmoregulation is fundamental for living cells and is particularly relevant for industrial biotechnology. Yeast adaptation to osmotic stress is an active process based on sensing and counterbalancing osmotic changes. Morphologic changes are key toward osmotic stress as yeast cells change their volume in response to osmotic challenges, decreasing volume in response to hypertonic stress and increasing volume in the presence of hypotonic stresses. Therefore, the ability to tolerate osmotic stress is strongly influenced by plasma membrane permeability (Gonzalez et al., 2016).

Through an adaptive laboratory evolution experiment, Zhu et al. (2020) were able to isolate a strain with improved tolerance to osmotic stress. Transcriptome sequencing (RNA-seq) suggested that mRNA levels of *ELO2* were differentially upregulated in the isolated strain. Using a reverse engineering strategy, *ELO2* was overexpressed in a wild-type strain, resulting in enhanced very long fatty acids content (the contents of C_20__:__0_, C_22__:__0_, and C_24__:__0_ were increased by 52.3, 94.1, and 14.4%, respectively). Furthermore, the levels of complex sphingolipids were increased. These modifications have been reported to promote a thicker and less permeable membrane. Flow cytometry analysis of cells stained with SYTOX green revealed that the *ELO2* overexpressing strain exhibited a 24.4% higher membrane integrity than the wild-type strain, resulting in an enhanced osmotic tolerance.

Similarly, Yin et al. (2020) were able to increase the tolerance of *S. cerevisiae* to salt stress by significantly improving the yeast membrane potential and integrity. Using a nitroguanidine mutagenesis strategy, authors identified *CDS1*, encoding a phosphatidate cytidylyltransferase, and *CHO1*, encoding a phosphatidylserine (PS) synthase, as key factors to yeast tolerance toward salt stress. The combined overexpression of *CDS1* and *CHO1* resulted in a redistribution of membrane phospholipids and a decreased anionic-to-zwitterionic phospholipid ratio. In *S. cerevisiae*, anionic phospholipids are mainly PA, PI, and PS, while zwitterionic phospholipids are PE and PC. These results indicate that a higher presence of zwitterionic phospholipids may be beneficial to deal with salt stress (Yin et al., 2020).

These works highlight that different membrane compositions can be advantageous toward the same stress agent. In both works, Yin et al. (2020) and Zhu et al. (2020), the levels of PS and PE were increased, 35.5% and 15, 25.2, and 18.9%, respectively. However, when *ELO2* was overexpressed the levels of PC, PA, and PI did not change. On the other hand, the double overexpression of *CDS1* and *CHO1* led to a 28.6% increase in PC and to a decrease in the levels of PA and PI of 14.6 and 39.8%, respectively.

### Enhancing the Production Capacity of Microbial Cell Factories: When Membrane Composition Can Contribute to Increasing Product Export

Microorganisms can be exploited for the production of several compounds with applications in a wide range of industrial sectors, and whenever possible the export of the product into the medium is preferable, mainly for limiting the downstream processing costs, but very often also for increasing the flux toward the product itself (Chung et al., 2015; Tsuge et al., 2016; Porro and Branduardi, 2017). Therefore, a proper export of the products of interest is often indispensable for a profitable and efficient cell factory. Researchers have been focusing on maximizing the export of the compounds of interest by overexpressing specific membrane transporters, engineering transporters for improved efficiency or even introducing heterologous transporters in the cell factory (Boyarskiy and Tullman-Ercek, 2015; Kell et al., 2015; Erian et al., 2020; Soares-Silva et al., 2020). For more detailed information on the engineering of membrane transporters for industrial biotechnology applications, the reader should look the works mentioned above.

However, many times, the simple overexpression of a transporter does not result in the intended increased export rates. This is why in this review we wanted to focus on and highlight strategies where the plasma membrane composition was changed, and in turn influencing the properties of the entire structure, considered as a system. Indeed, the plasma membrane composition is very crucial and must be considered since it determines physiochemical properties such as fluidity, permeability and elasticity, which can facilitate the export of the products of interest (Royce et al., 2015). So far there are not many examples of membrane engineering strategies focusing on increasing metabolites secretion in yeast and in particular in *S. cerevisiae*. However, there are many works performed in *E. coli* (Tan et al., 2016, 2017; Wu et al., 2017; Kanonenberg et al., 2019) which may serve as guidance for future work in yeast.

One interesting example in *S. cerevisiae* relates to the work of Liu et al. (2016) who reported a decrease in the fluidity of the plasma membrane caused by the decrease of unsaturated fatty acids in *S. cerevi*siae strain producing β-carotenes. Carotenoids accumulate in the cell membrane and therefore, high production levels of carotenoids can cause membrane stress (Gruszecki and Strzałka, 2005).

In this strain, carotenoid biosynthesis shares the precursors acetyl-CoA and farnesyl pyrophosphate (FPP) with unsaturated fatty acids and ergosterol, respectively. Therefore, heterologous carotenoid biosynthesis could decrease the content of unsaturated fatty acids and ergosterol due to competition for these precursors. Given the importance of plasma membrane fluidity in cellular metabolism and physiology (such as facilitating the absorption of essential substances), authors sought to restore it. The addition of linoleic acid (C_18__:__2_) to the culture media restored the plasma membrane fluidity by the incorporation of unsaturated fatty acids in the membrane. This resulted in a 24.3% increase in the production of β -carotene (Liu et al., 2016).

A different approach was used by Bu et al. (2020). The authors were able to counteract the decrease of membrane fluidity caused by the accumulation of β-carotenes by overexpressing *OLE1*. Overexpression of *OLE1* could improve the fatty acid unsaturation and membrane flexibility, which conferred cells a high tolerance to various types of stress, as reported above (Fang et al., 2017; Nasution et al., 2017). Indeed, the overexpression of *OLE1* promoted cell membrane fluidity (measured by fluorescence anisotropy) and resulted in an improved β-carotene secretion (Bu et al., 2020). The same strategy was also used by Hong et al. (2019) for the production of lycopene, a red carotenoid pigment. *OLE1* overexpression led to improved lycopene production suggesting that an increase in unsaturated fatty acids content in the cell membrane might relieve the carotenoid toxicity (Hong et al., 2019).

Another example of how the membrane composition affects the export of molecules was reported by Wang et al. (2013). The addition of surfactants, such as Triton X-100, led to increased permeability and fluidity of the plasma membrane in the yeast *Monascus purpureus*, which resulted in a 56.8% higher production of pigments. The addition of Triton X-100 led to an increased degree of unsaturation in the membrane lipids. According to the authors, these changes, facilitated the secretion of intracellular pigment to the broth thus alleviated the product feedback inhibition and enhanced pigment production (Wang et al., 2013). These results suggested that Triton X-100 could markedly affect the fatty acid composition of *M. purpureus* H1102 by significantly increasing the degree of unsaturation of the cell membrane lipids, thus improving the fluidity and permeability of the cell membrane.

## Conclusion

Overall, despite its great potential, membrane engineering is still a highly complex approach as membrane lipids and membrane homeostasis are vital for many cellular functions. Moreover, it is difficult to predict the outcomes of altering membrane elements in the whole membrane system. The understanding of membranes and their structure has changed enormously over the last years. The availability and development of high-throughput methods have allowed researchers to deepen their knowledge on the plasma membrane conformation and dynamics. However, the analysis of the plasma membrane composition is still challenging which hampers a detailed association between specific composition and physicochemical properties of the plasma membrane. The interdependency between membrane lipids and proteins can not be neglected. In the future, a combination of efforts between researchers from different areas of study, such as lipidomics, molecular dynamics simulations and membrane biophysics will be crucial to gain a better understanding of the plasma membrane and therefore plan strategies to tailor it at a systems level.

## Author Contributions

LF and PB drafted the manuscript. LF wrote the manuscript. MS, MJS, and PB provided the writing guidance and revised the manuscript. All authors read and approved the final manuscript.

## Conflict of Interest

The authors declare that the research was conducted in the absence of any commercial or financial relationships that could be construed as a potential conflict of interest.

## Publisher’s Note

All claims expressed in this article are solely those of the authors and do not necessarily represent those of their affiliated organizations, or those of the publisher, the editors and the reviewers. Any product that may be evaluated in this article, or claim that may be made by its manufacturer, is not guaranteed or endorsed by the publisher.
